# Investment in human resources improves eye health for all

**Published:** 2018-07-31

**Authors:** Daksha Patel, Suzanne Gilbert

**Affiliations:** 1 E-learning Director: International Centre for Eye Health, London School of Hygiene and Tropical Medicine, London, UK.; 2 Senior Director: Innovation & Sight Programs, Seva Foundation, Berkeley, California USA.

**There is a widening gap between the need for eye health workers and their availability in low-income countries. Change will come once governments recognise the health workforce as a productive investment, not an expense.**

**Figure:**
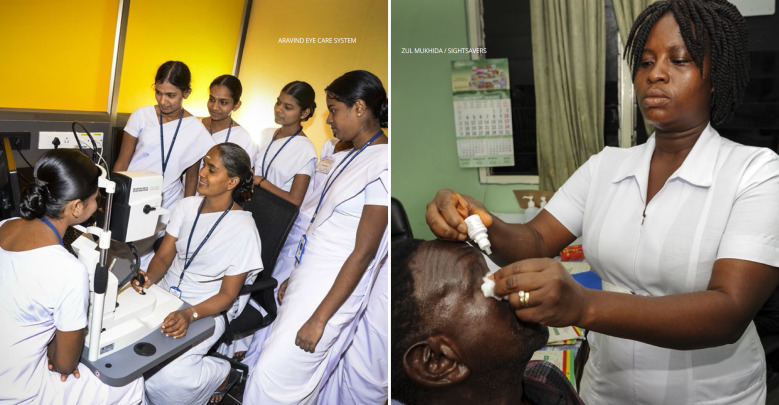
Trainees learn about OCT machines. INDIA A qualified ophthalmic nurse instils eye drops. GHANA

Worldwide, we face a substantial and widening gap between the number and types of health workers needed to provide essential services, and both their availability, and governments' capacity to employ them. This is especially true in low- and middle-income countries. In order to achieve universal health coverage, including meeting the needs of a growing and ageing population, an estimated 54.5 million health workers will be required by 2030. In low-income countries, this means the supply of health workers will have to increase by 40%.^1^

In eye health, the situation is much the same. A paper published by the Vision Loss Expert Group^2^ forecasts that there will be close to a three-fold increase in the number of people with visual impairment over 35 years: from 252 million in 2015 to 702 million in 2050. This threatens to overwhelm existing levels of service provision. It will be a challenge to train and deploy a sufficient number of eye health workers. For example, in Sub-Saharan Africa, there is currently only half the minimum number of eye health professionals recommended by the World Health Organization.

## A paradigm shift

Governments must recognise the health workforce as a productive **investment** instead of an expense.^3^ The objectives set out in WHO's Global Strategy on Human Resources for Health: Workforce 2030,^1^ describes human resources for health as an investment that enables an ‘improvement in health outcomes, social welfare, employment creation and economic growth.’

Investment in trained eye health personnel is essential in order to reduce avoidable blindness and visual impairment, which not only transforms individual lives for the better, but also improves productivity and reduces costs at national level.^4^

## Challenges

In order to improve the availability, retention and productivity of eye health personnel, it is important to understand the current situation and what needs to be done.

### Availability: number and distribution

Globally, as well as within regions and countries, the eye health workforce is unequally distributed, with poorer areas – where the need for eye care services is highest – receiving the least coverage. Worryingly, training is also unequally distributed. A comprehensive study of data from 193 countries^5^ estimated that, based on the training facilities currently available, the number of ophthalmologists being trained worldwide is insufficient to keep pace with population growth trends. This is worst in low-income countries, where just 1.7 resident ophthalmologists per million are being trained annually, compared to 8.5 residents per million in high-income countries.

It is important to plan human resources for eye health based on local needs. Investment in ophthalmic training for every member of the eye team, and across all service levels, is needed, and this requires an innovative, nationally relevant approach. For example, populations on the Indian sub-continent have a higher incidence of cataract than those in Sub Saharan Africa, a difference that must be taken into account when training, recruiting and distributing eye care workers.

### Productivity

The productivity of the eye health workforce is affected by the availability of well functioning infrastructure and equipment. Such essential investment supports staff motivation and high quality eye care delivery. However, it is important to recognise that productivity in some low-income settings will be affected due to the combined pressures of a high need for eye care, limited access due to distance and cost, inadequate infrastructure, and legal restrictions e.g. those that prevent non-ophthalmologists to perform cataract surgery or undertake refraction. Tools for planning the health workforce, such as the World Health Organization's Workload Indicators of Staffing Needs (**www.who.int/hrh/resources/wisn_user_manual/en**), are being introduced. Where possible, it would be beneficial to explore integrating eye health workforce planning within these tools.

### The team approach

The team approach is an important component of productivity. In cataract surgery, for example, it is widely acknowledged that it can improve both efficiency (the number of operations per surgeon) and effectiveness (the quality of surgery). The team approach works by ensuring that ophthalmologists – a scarce resource – do not perform tasks which can be done by other staff members once they are trained and qualified. This is known as ‘task shifting’ and is very important in improving productivity and job satisfaction.

### Retention

Retention involves encouraging trained and experienced eye health workers to remain in post. The rate of attrition (a loss of personnel for reasons other than death or retirement) vary between countries and among the different types of eye care professionals. For example, the attrition rate of trichiasis surgeons has been found to be as high as 59% in Northern Ethiopia; this is linked with poor mobile phone coverage, electricity supply, and road access.

## Moving forward

Before investing in new strategies and policies, such as those suggested in Table 1, it is important to gather evidence, consider local needs and resources, and examine the effectiveness of past efforts. In this issue, we have included several short case studies and perspectives which we hope will serve as a useful starting point towards our common goal of providing universal access to eye health worldwide.

**Table 1:** Strategies for improving the availability of human resources for eye care

	Investment requirements: financial and non-financial
Training (p. 41–43)	Appropriate localised selection criteria for training Competency-based curriculums Expansion of training programmes Greater flexibility and access to training, e.g. distance education, flipped classroom or blended learning Support lifelong learning resources and opportunities for all eye health personnel
Training and managing eye teams (pp. 41–43 and 51–52)	Identification and training for the range of competencies required. Nationally specific structured career development for mid-level personnel. Task shifting and task sharing to bridge gaps and ensure the team has the right mix of skills
Recruitment and distribution (pp. 45–47)	Plan for needs-based training Plan distribution targets at all levels (community, primary, secondary and tertiary) Policies across promotive, preventive, curative, and rehabilitative services
Retention, motivation and management (pp. 48–52)	Appropriate remuneration Improved facilities and equipment Supportive management and leadership CPD and other career development opportunities Supervision for Allied Ophthalmic Personnel
Advocacy	All eye health stakeholders to unite around resolving the workforce crisis Introduce integrated workforce planning which is supported by resource allocation Strengthen data and evidence for the cost/benefit of human resource development as an investment.
